# Research on Migration and Surface Deformation during Goaf CO_2_ Geological Storage—Taking Mindong No.1 Mine in Hulunbuir as an Example

**DOI:** 10.1021/acsomega.6c03093

**Published:** 2026-08-24

**Authors:** Qiang Liu, Yuan Li, Chaoyue Huang, Jialong Li, Bing Liang, Weiji Sun, Jiaxu Jin, Jianjun Liu, Hu Li

**Affiliations:** † State Key Laboratory of Geomechanics and Geotechnical Engineering Safety, Institute of Rock and Soil Mechanics, Chinese Academy of Sciences, Wuhan 430071, China; ‡ School of Mechanics and Engineering, 91588 Liaoning Technical University, Fuxin 123000, China; § Ordos Institute of Liaoning Technical University, Ordos 017004, China; ∥ School of Civil Engineering, Liaoning Technical University, Fuxin 123000, China; ⊥ 74601 Sichuan University of Science and Engineering, Yibin 644000, China; # BJCM Deep Earth Energy and Environmental Science Research Institute, Beijing 102206, China

## Abstract

Goaf CO_2_ geological storage is a key technology for addressing the dual issues of the utilization of large-scale coal mine goaf and the space for CO_2_ storage. Exploring the migration range of CO_2_ and the surface deformation during the storage process is the core challenge in ensuring the safety and stability. Based on the geological conditions of Mengdong Energy’s Mindong No.1 mine in Hulunbuir, a real geological structure model of the goaf, overburden of goaf, caprock, and overburden was established, and numerical simulation experiments on CO_2_ migration and surface deformation were conducted. The research results indicate that a higher storage pressure led to a larger CO_2_ migration range and a faster migration rate. After 20 years of storage, the amount of CO_2_ diffused from the overburden to the surface was consistently below 0.02% under all pressures investigated, and no significant leakage was observed. The amount of CO_2_ diffused in the first five years exceeded 50% of the total migration over the 20 year period. In contrast, the amount diffused in the last five years was less than 7.4% of the total. In the first year of storage, pressure was identified as the dominant force driving CO_2_ migration. By the twentieth year, the pressure difference had diminished, and the various forces mutually counteracted each other. Consequently, CO_2_ migration nearly ceased. During the process of CO_2_ storage in the goaf, the surface deformation is characterized by an initial uplift followed by a progressive decline from the peak-uplift position, while the surface remains above its preinjection level. Under the storage pressures of 10, 15, and 20 MPa, the maximum uplift amounts are 0.060, 0.0917, and 0.123 m, respectively. The evolution can be divided into three stages: rapid uplift-decline period, slow adjustment period, and stabilization period. The decline in uplift is mainly governed by changes in effective stress: at the initial stage of storage, the pore pressure is the highest and the effective stress is the lowest, and the surface uplift reaches the peak; when the pore pressure decreases and the effective stress increases, compression of the strata causes the surface displacement to decline from its peak-uplift position. After 20 years of storage, the Surface-uplift recovery ratios under the storage pressures of 10, 15, and 20 MPa are 27.48%, 14.99%, and 9.76%, respectively. The higher the storage pressure, the smaller the reduction from the peak uplift and the lower the recovery rate. These findings confirm the target strata’s suitability for long-term CO_2_ storage.

## Graphical abstract

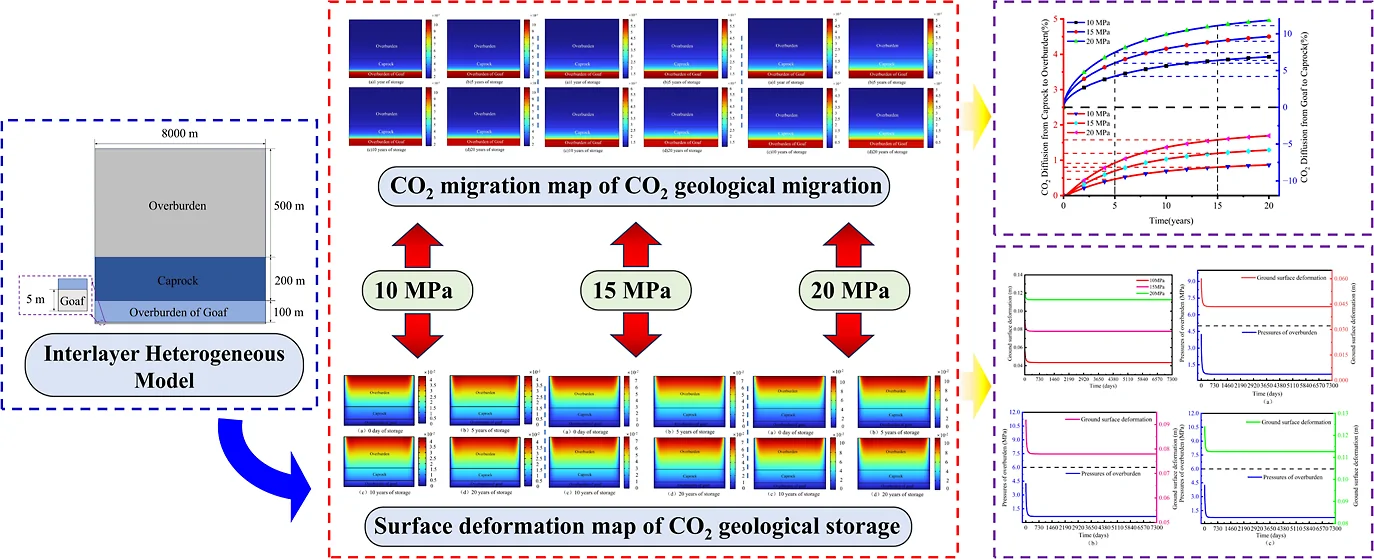

## 1 Introduction

Continued coal mining has resulted in extensive mined-out areas.^1^ It is estimated that by 2030, the underground space will reach 23.452 billion cubic meters.^2^ These areas are prone to inducing geological disasters such as residual coal spontaneous combustion, surface subsidence, and seismic events.^3–5^ Meanwhile, in the context of global efforts to promote carbon emission reduction, integrating the CO_2_ geological storage technology (Carbon Storage) with the management of abandoned mining areas can not only efficiently utilize underground space but also contribute to carbon reduction, forming a sustainable development path of “using waste to solve problems and achieving a green cycle”.^6–10^ Compared with CO_2_ storage in coal seams, goaf-based storage offers several potential advantages. In coal seams, CO_2_ is stored primarily through adsorption within the coal matrix, and the storage performance is strongly influenced by coal rank, porosity, water content, and permeability.^11^ In contrast, the extensive void space created by coal extraction can provide substantial storage capacity, while the relatively high permeability of the caved zone can improve CO_2_ injectivity and facilitate its migration and redistribution.^12^ Therefore, storing CO_2_ in underground goafs not only enables the reuse of abandoned mine space but also achieves effective storage of CO_2_. Successful CO_2_ geological storage must achieve the target storage capacity while strictly adhering to two “safety red lines”: (1) Ensure that the storage body is sealed to prevent the CO_2_ leakage. (2) Effectively control the reservoir pressure to avoid disturbing the geological structure, thereby ensuring the stability of the surface form and the safety of the ground facilities.

Even when the CO_2_ geological storage technology is applied, the gas may still break through this crucial barrier of the caprock and leak to the shallow area, posing a potential risk of surface deformation.^13–18^ Therefore, the sealing integrity of the caprock must be given high priority. A large number of studies had shown that CO_2_ has a significant impact on reservoir rocks. Ranathunga et al.^19^ investigated the changes in the physical, chemical structure, and mechanical properties of rocks after CO_2_ adsorption and concluded that CO_2_ causes the mechanical properties to weaken and the compressive strength to decrease. Hu et al.^20^ pointed out that as the gas pressure increases, the compressive strength of the rock decreases. The reason lies in the expansion stress and chemical erosion, which jointly weaken the interparticle force in the rock and reduce their surface energy. Li et al.^21^ and Choi et al.^22^ conducted rock-mechanics experiments before and after the action of CO_2_. The test results indicated that the elastic modulus and compressive strength of the rock were decreased, and its mechanical properties notably were deteriorated. Ozotta et al.^23^ used nanoindentation to detect the mechanical changes in rocks before and after CO_2_ injection. The results showed that the long-term action of CO_2_ led to the loss of rock, decreased the elastic modulus, and increased the Poisson’s ratio. In terms of caprock integrity, Ranjith et al.^24^ found through acoustic emission monitoring that for the rock samples after being subjected to 1 MPa CO_2_ pressure for 72 h, the stress during the crack closure and initiation stages accounted for a higher proportion of the peak strength. Shukla et al.^25^ summarized the mechanism of damage to caprock integrity and concluded that fault shear failure and capillary pressure overpressure were the most significant factors. Li et al.^26^ conducted a CO_2_ immersion experiment and found that as the immersion time increased, the tensile strengths of the rock matrix, the in-layer normal planes, and the interlayer rock interfaces all showed a downward trend. Furthermore, the phase state of CO_2_ and the relationship between rock microdamage and mechanical properties were comprehensively considered by Li et al.,^27^ and the experiment of CO_2_ immersion in gaseous/supercritical states was conducted. The results indicated that the main factors causing the deterioration of mechanical properties were the increase in pore volume and the increase in interconnected pores.

In addition, during the process of CO_2_ geological storage, the changes in reservoir pressure are the dominant factors causing alterations in the stress field of deep rocks. These changes will propagate upward and directly determine whether deformation phenomena such as uplift or subsidence occur on the earth surface.^28–31^ Rahman^32^ adopted the fluid–solid coupling method to analyze the fluid pressure and stress–strain changes in the CO_2_ geological reservoir. It was found that when the storage volume of CO_2_ was large, the maximum surface uplift could reach 4.3 cm. Based on this, the method was upgraded by Yin et al.,^33^ and a thermal-structural-fluid three-field coupling analysis method by considering the influence of temperature was established. Rutqvist^34^ observed through the study of CO_2_ injection formations that the surface elevation rose by approximately 5 mm per year above the active CO_2_ injection wells, and the elevation pattern extended outward several kilometers from the injection wells. In summary, the current research on CO_2_ geological storage leakage and surface deformation mainly focuses on typical reservoir media such as deep saline aquifers and unextractable coal seams. However, in large-scale mined-out areas, the unique leakage and surface deformation patterns have not been systematically studied, and they remain key scientific issues that urgently need to be clarified in this field.

The existing storage methods mainly include deep saline aquifer storage, depleted oil reservoir storage, and unmineable coal seam storage. These storage methods are affected by factors such as storage space and storage pressure, making it difficult to store CO_2_ effectively. Therefore, this manuscript innovatively explores the geological storage of CO_2_ in coal mine goaf. In response to the key scientific issues regarding the unclear leakage and surface deformation laws during the CO_2_ storage in goaf, the effects of different storage pressures and times were comprehensively considered. A numerical model of the geological structure of the goaf, overburden of goaf, caprock and overburden was established, and numerical simulation studies on CO_2_ storage—migration—surface deformation were conducted. The influence of different storage pressures and times on CO_2_ migration was revealed, and the surface deformation laws under CO_2_ geological storage in the goaf were clarified. The main novelty lies in establishing a field-scale “goaf–goaf overburden–caprock–upper overburden” model using site-specific measured geological parameters, investigating CO_2_ migration and surface deformation during the postinjection storage stage, and providing insights into the pressure–migration–deformation relationships relevant to CO_2_ storage in other coal mine goafs. This aims to provide theoretical basis and scientific evidence for evaluating the long-term storage stability and safety of CO_2_.

## 2 Numerical Model Construction

### 2.1 Characterization of the Geological Structure Model

On the basis of actual test data from field parameter wells, a stratum without faults was selected as the study object to better ensure the storage security and stability of CO_2_ storage in the goaf. A field-scale geological structure model was established to simulate the CO_2_ leakage and surface deformation during the storage process. On the basis of the actual geological conditions of the Mindong No.1 mine, a field-scale realistic geological structure model with a width of 8000 m and a depth of 805 m was established. Considering the heterogeneity among different strata, the model was divided into four layers: goaf, overburden of goaf, caprock, and overburden, as shown in Figure 1. The thicknesses of the goaf, overburden of goaf, caprock, and overburden are 5 m, 100 m, 200 m, and 500 m, respectively. Core samples of the overburden of goaf, caprock, and overburden were drilled on-site, and the key parameters including porosity, permeability, and elastic modulus were measured. The present study mainly focuses on two aspects: whether CO_2_ leakage occurs in the goaf and the pattern of surface deformation rather than on the CO_2_ leakage pathways in the goaf. Moreover, the target formation was deliberately selected as a suitable storage formation, without large-scale fractures. Therefore, the fracture system was neglected in the model construction. The basic parameters of different strata are listed in Table 1.

**Figure 1:**
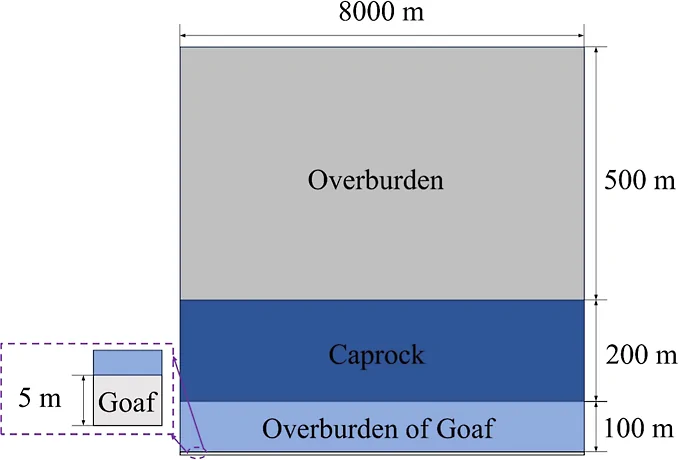
Numerical model of the goaf-overburden of the goaf-caprock-overburden geological structure.

**Table 1:** Basic Parameters of Different Strata

strata	thickness (m)	porosity (%)	permeability (md)	elastic modulus (GPa)	Poisson’s ratio	density (kg·m^–3^)	Biot coefficients
overburden	500	1	0.01	6	0.2	2600	0.2
caprock	200	1	0.01	15	0.15	2700	0.2
overburden of goaf	100	25	50	10	0.2	2400	0.5
goaf	5	-	-	-	-	-	

### 2.2 Theory of Multiphase Darcy Flow in Porous Media

Darcy’s law, as the fundamental law describing the flow of fluids in porous media, has significant advantages in dealing with fluid flow. In previous studies, the influence of pore structure heterogeneity of core samples from this formation was investigated at the microscale and mesoscale.^14,16^ To simulate whether CO_2_ leakage occurs during CO_2_ storage at the field scale and the resulting surface deformation effects, and to lay a foundation for subsequent research on establishing an accurate field-scale heterogeneous and anisotropic model. Because the lithology within each stratum is relatively consistent and no large-scale fractures or fault systems are present, each layer was assumed to be homogeneous and isotropic. In this study, The “Darcy’s Law” physics field in the “Porous Media and Groundwater Flow” module of the COMSOL Multiphysics software was employed. The simulation adhered to the following basic assumptions:

(1) Fluid flow in the porous media conformed to Darcy’s law;
(2) The two fluid phases were assumed to be immiscible.
(3) The entire simulation process was considered an isothermal system.
(4) The porous media were assumed to be homogeneous and isotropic.

The basic governing equations for the two-phase Darcy flow model consisted of an extended Darcy’s law and a continuity equation. The saturation variable was introduced for coupling. The governing equations were formulated as follows:^35–37^

The mass conservation equation and the Darcy equation were given by

1 $$\frac{\partial }{\partial t}\left(\phi p\right)+\nabla \cdot \left(pu\right)=Q_{m}$$

2 $$u=-\frac{\kappa }{\mu }\left(\nabla p+pg\nabla d\right)$$

where *p* is pore pressure, Pa; **u** is the Darcy velocity vector, m/s; φ is porosity, dimensionless; *Q*_m_ is the source term; *K* is permeability, md; μ is fluid viscosity, Pa·s; *d* is the storage depth in the gravity direction, m.

The porous elastic theory and the effective stress principle were introduced to accurately simulate surface deformation caused by CO_2_ storage. This approach fully reflected the solid deformation caused by pore pressure changes and the feedback effect of deformation on the fluid flow field.^38–40^ The stress equilibrium equation, the stress–strain equation, and the strain–displacement equation used for simulating the deformation are given as follows

3 ∇·σ+F=0

4 $$\sigma^{\prime }=\sigma -\alpha pI$$

5 $$\sigma^{\prime }=2\frac{E}{2\left(1+\nu \right)}\varepsilon +\lambda tr\left(\varepsilon \right)I$$

6 $$\varepsilon =\frac{1}{2}\left[\nabla u^{\prime }+{\left(\nabla u^{\prime }\right)}^{T}\right]$$

where **σ** is the total stress tensor, N/m^2^; **F** is the body force vector, N/m^3^; ***σ′*** is the effective stress tensor, N/m^2^; *E* is the elastic modulus, Pa; ***v*** is Poisson’s ratio, dimensionless; α is the Biot coefficient, dimensionless; *p* is pore pressure, Pa; ***ε*** is the strain tensor, dimensionless; **I** is the identity matrix, dimensionless; **u′** is the displacement vector, m.

### 2.3 Fluid Model Construction and Boundary Conditions

The target storage area to be sealed is the goaf. Because the overburden of the goaf has relatively high porosity and permeability and may be affected by the three-zone structure and fractures induced during mining, its sealing capacity is weaker than that of the intact caprock and overlying strata. Therefore, the sealing effect of the goaf overburden was not considered. Instead, this study focused on the sealing performance and deformation behavior of the caprock and overlying strata. The numerical simulation was initiated from the condition in which CO_2_ in the goaf had already fully filled the overburden of the goaf, thereby representing the worst initial state after injection, where the local space was completely occupied by CO_2_. Therefore, only CO_2_ migration in the overburden of goaf, caprock, and overburden need to be considered. In the overburden of goaf, the initial CO_2_ saturation was set to 100%, and the water saturation was set to 0%. In the caprock and overburden, the initial CO_2_ saturation was set to 0%, and the water saturation was set to 100%. Based on the formation pressure and temperature gradients, combined with field measurement data, the pressure and temperature of the target reservoir (overburden of goaf) were 8 MPa and 50 °C, respectively. The critical temperature and pressure for supercritical CO_2_ (ScCO_2_) are 31.4 °C and 7.38 MPa. Therefore, the CO_2_ storage in the target formation was in a supercritical state. The top boundary represents the contact interface between the ground surface and the atmosphere. Therefore, a pressure of 0.1 MPa was assigned to characterize the atmospheric pressure above the surface. The selection of storage pressures was based on a comprehensive consideration of the initial pressure of the target formation, the conditions required to maintain CO_2_ in a supercritical state, and the results of triaxial compression tests conducted on the overburden above the goaf. Based on the formation pressure gradient, the initial pressure of the target formation is approximately 8 MPa. As shown in Figure 2, triaxial compression tests were conducted on rock samples collected from the goaf overburden. The experimental results are presented in Table 2, at a confining pressure of 8 MPa, the specimens exhibited a triaxial compressive strength of 21.2 MPa, corresponding to a peak deviatoric stress of 13.2 MPa. These experimental results provide a basis for evaluating the deformation and shear stability of the goaf overburden under different pressure conditions. The initial parameters of water and CO_2_ in different strata are shown in Table 3.

**Figure 2:**
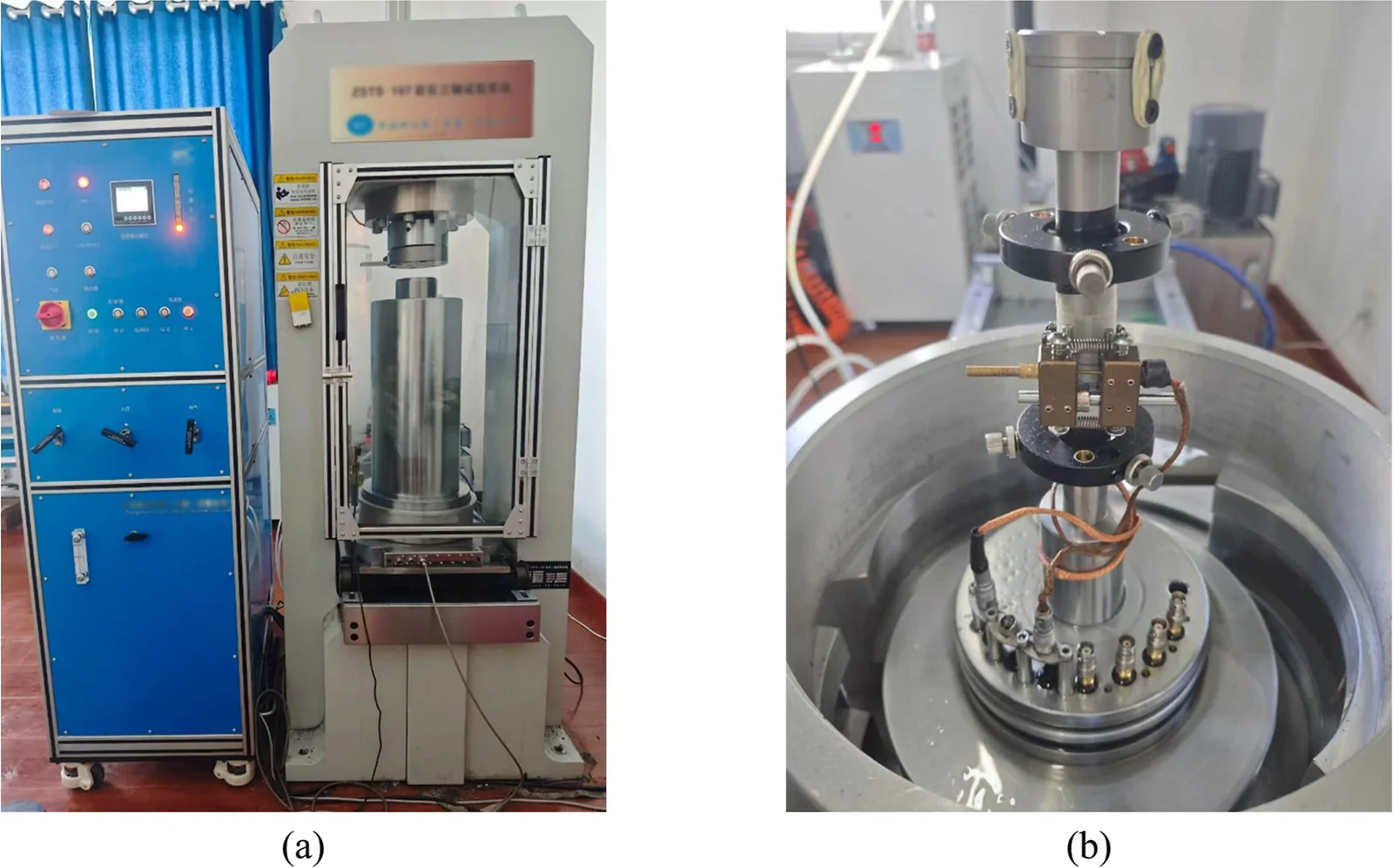
Triaxial compression test, (a) triaxial compression testing apparatus; (b) pressure chamber.

**Table 2:** Triaxial Compression Test Date

types of rocks (Φ25 cm × 50 cm)	confining pressure (MPa)	peak deviatoric stress (MPa)	triaxial compressive strength (MPa)
sandstone	8	13.2	21.2

**Table 3:** Initial Parameters of Water and CO_2_ in Different Strata

parameter	water	CO_2_ at different pressures		
initial pressure (MPa)	–0.0011*y* + 10	10	15	20
temperature (K)	–0.03*y + 323.15	323.15	323.15	323.15
density (kg/m^3^)	1100	211.96	577.53	713.46
viscosity (mPa·s)	1.5 × 10^–2^	2.70 × 10^–2^	3.83 × 10^–2^	5.02 × 10^–2^

The boundary conditions for the model were defined as follows:

(1) The boundaries on both sides and the bottom of the model were set as fixed constraints with no flux. Lateral displacement was restricted to the sides.
(2) The top boundary was set as an outlet with a pressure of 0.1 MPa to simulate the influence of the atmospheric pressure on storage and surface deformation. This boundary was also set as a free boundary to observe the deformation.

## 3 Results and Discussion

### 3.1 Influence of Different Storage Pressures on CO_2_ Migration

To investigate the CO_2_ migration law in the goaf under different injection pressures and clarify the synergistic mechanism between storage pressure and CO_2_ migration, numerical simulation studies were performed for storage periods of 1 year, 5 years, 10 years, and 20 years at pressures of 10, 15, and 20 MPa, respectively.

The initial distribution of water and CO_2_ in each stratum is shown in Figure 3. Red represents the CO_2_ phase, and blue represents the water phase. The CO_2_ migration under different storage pressures and storage times is shown in Figures 4–6. Compared with the initial state, the residual CO_2_ concentration in the overburden of the goaf region gradually decreased over time under the same pressure. The most significant reduction in residual CO_2_ concentration occurred between the initial state and the first year of storage. The changes between 10 and 20 years of storage were relatively small. At the same storage time, the residual CO_2_ concentration decreased as the pressure increased. In the caprock region, upward CO_2_ migration from the overburden of the goaf became more evident with increasing storage time under the same pressure. At the same storage time, the storage pressure significantly influenced the CO_2_ migration. A higher storage pressure resulted in a greater degree of upward migration of CO_2_. In the overburden region, no significant CO_2_ migration was observed in the figures, regardless of increased time or pressure.

**Figure 3:**
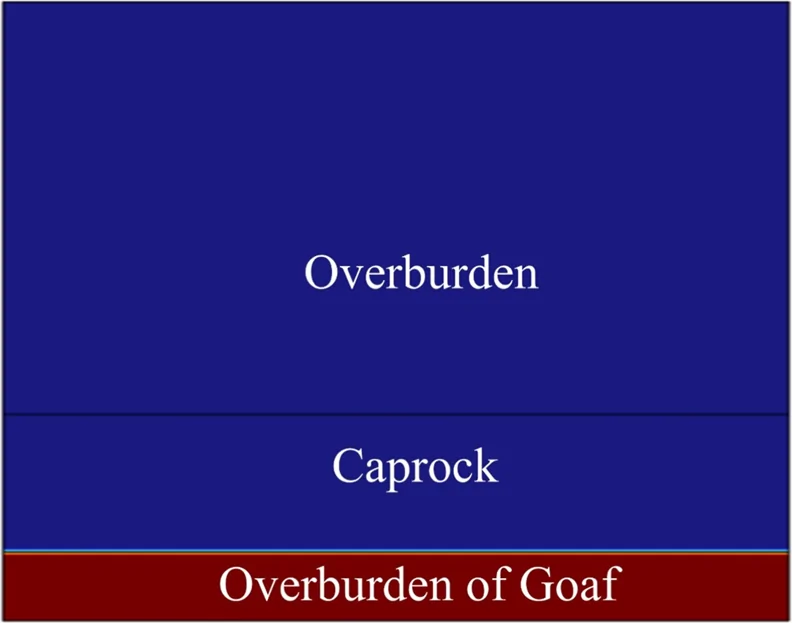
Initial distribution cloud of water and CO_2_ in each stratum.

**Figure 4:**
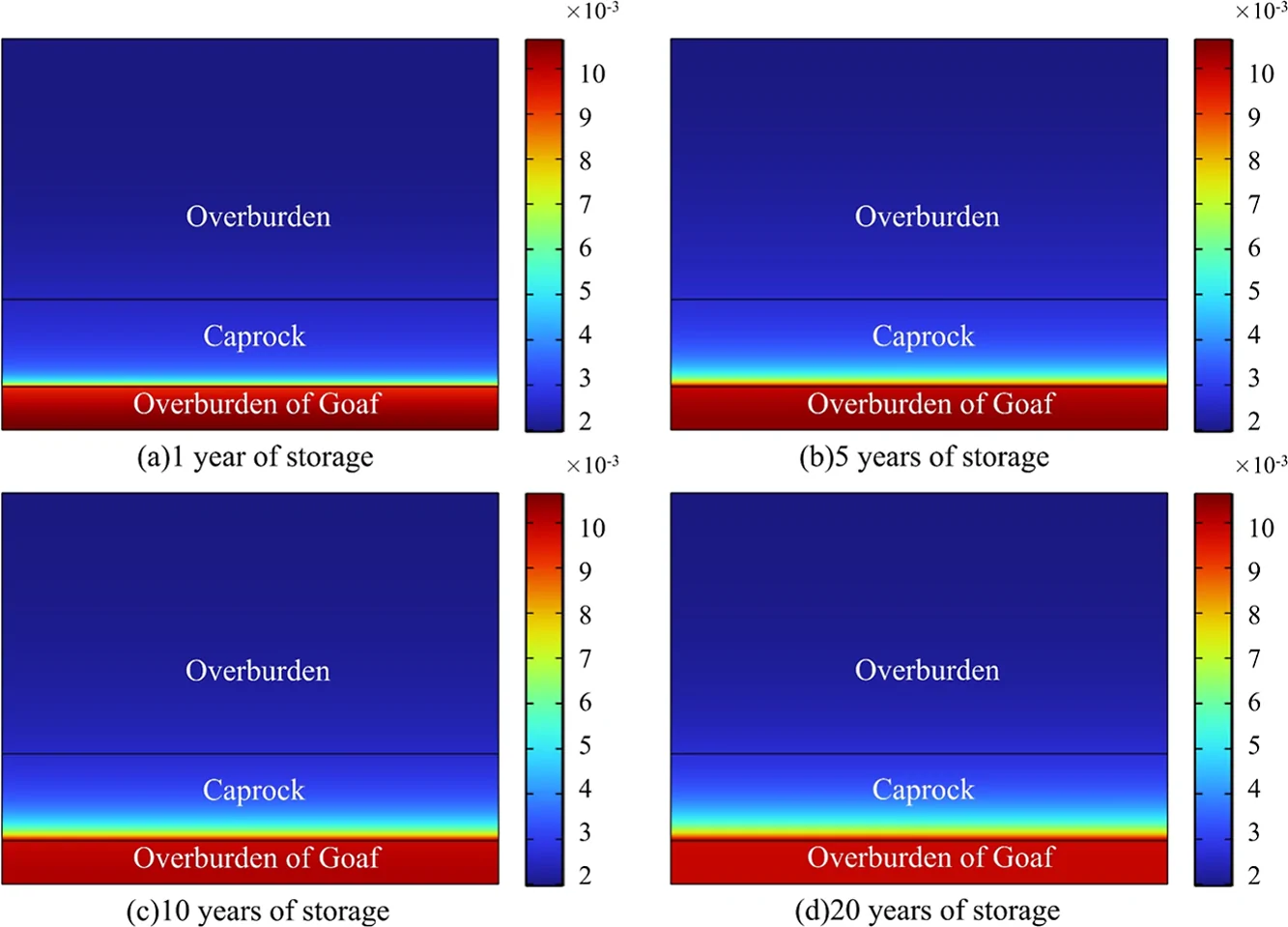
CO_2_ migration cloud at different storage times under 10 MPa. (a) One year of storage; (b) five years of storage; (c) ten years of storage; (d) twenty years of storage.

**5:**
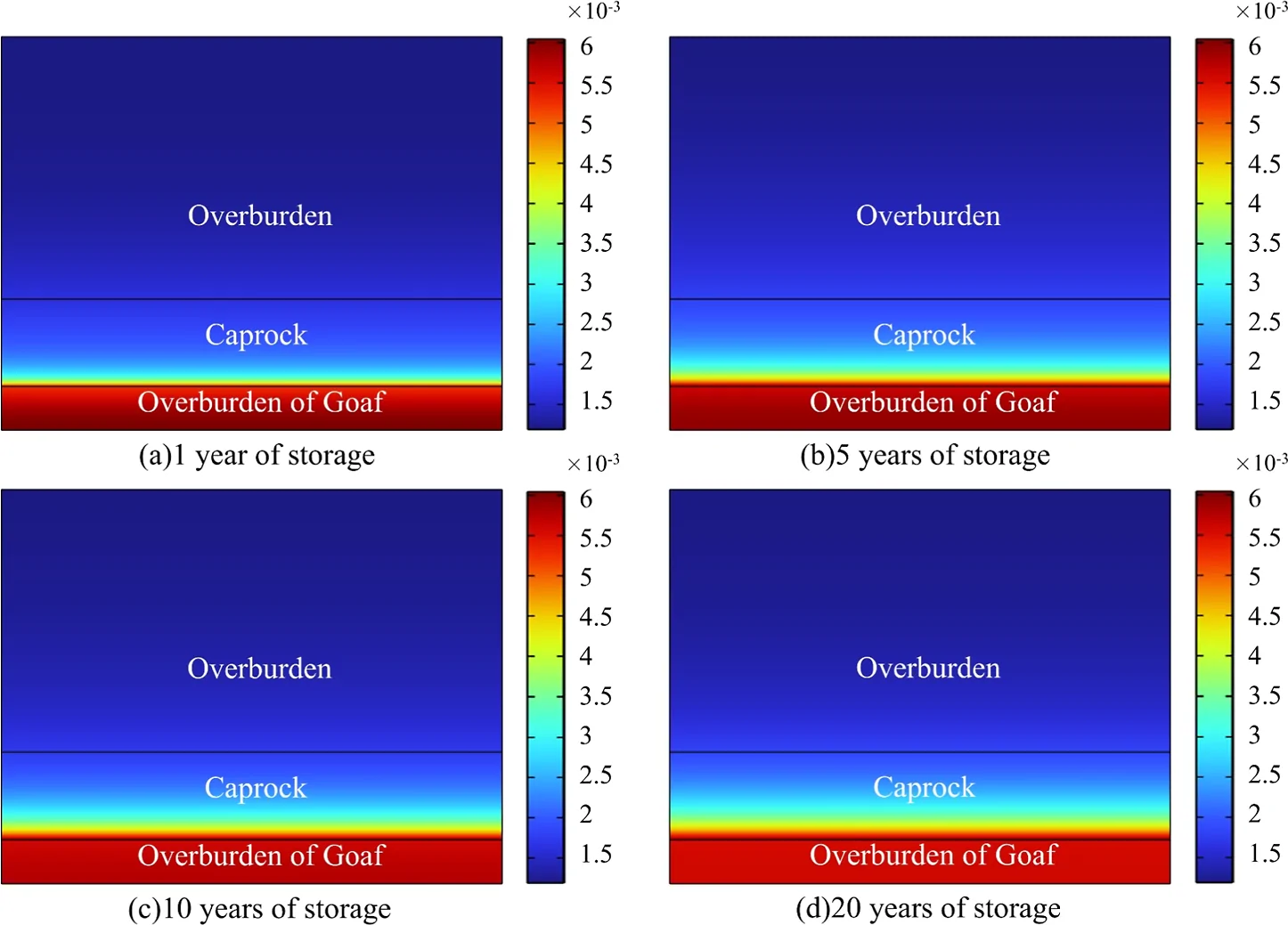
CO_2_ migration cloud at different storage times under 15 MPa. (a) One year of storage; (b) five years of storage; (c) ten years of storage; (d) twenty years of storage.

**Figure 6:**
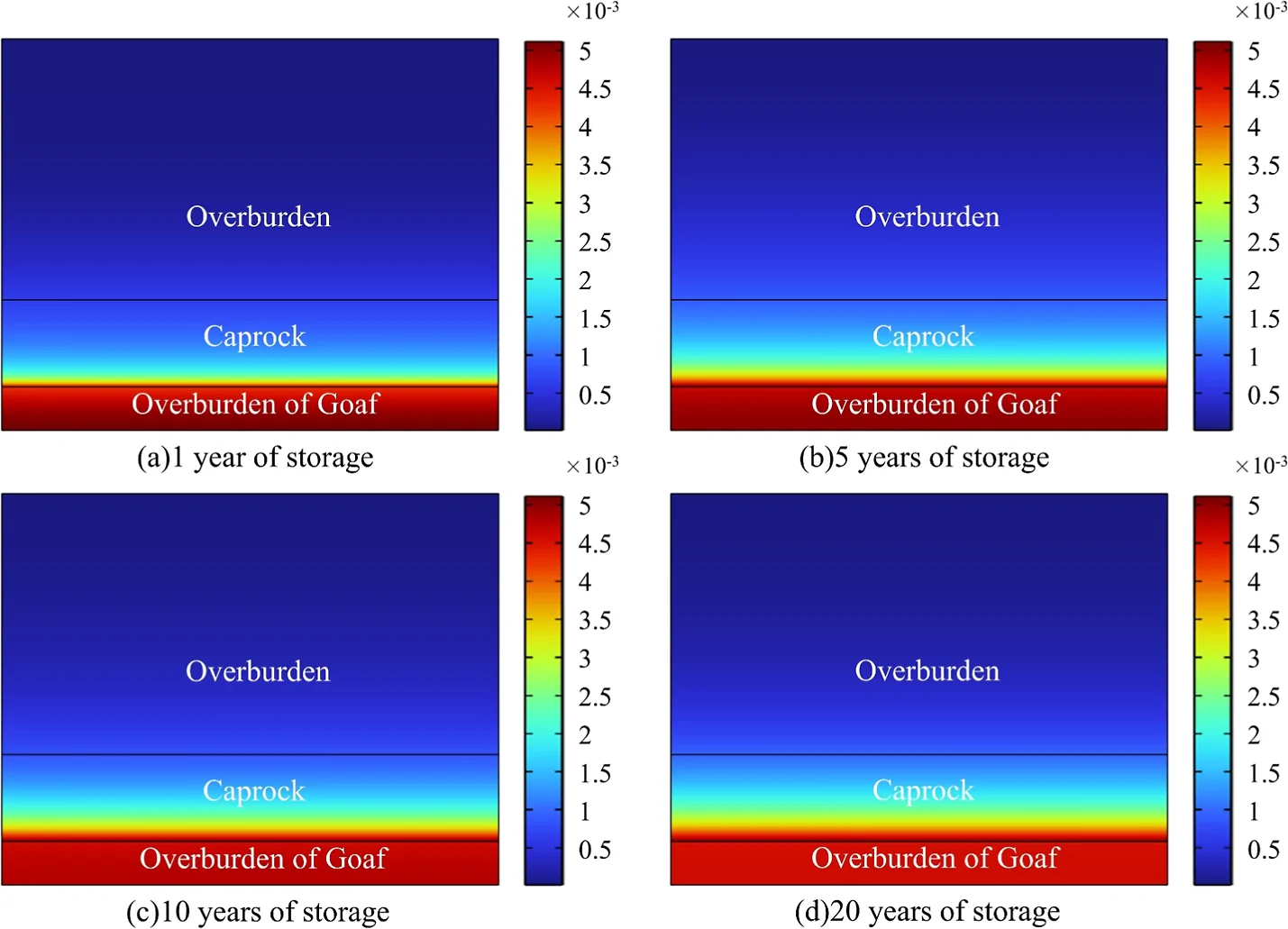
CO_2_ migration cloud at different storage times under 20 MPa. (a) One year of storage; (b) five years of storage; (c) ten years of storage; (d) twenty years of storage.

The migration amount of CO_2_ into each stratum over time under different storage pressures was calculated, and the results are presented in Table 4. The numerical simulation results indicated that the migration amount of CO_2_ from the overburden of the goaf into the caprock increased with higher storage pressure. Under a storage pressure of 10 MPa, the migration amounts after 1, 5, 10, and 20 years were 1.88%, 4.23%, 5.63%, and 6.87% of the total initial amount of CO_2_, respectively. Under 15 MPa, the migration amounts were 2.72%, 6.03%, 7.96%, and 9.61%, respectively. Under 20 MPa, the migration amounts were 3.29%, 7.47%, 9.88%, and 11.82%, respectively. The migration of CO_2_ from the overburden of the goaf into the caprock was enhanced as the pressure increased. For CO_2_ migration from the caprock into the overburden, under 10 MPa, the migration amounts after 1, 5, 10, and 20 years were 0.10%, 0.47%, 0.70%, and 0.87%, respectively. Under 15 MPa, the migration amounts were 0.15%, 0.70%, 1.03%, and 1.29%, respectively. Under 20 MPa, the migration amounts were 0.20%, 0.93%, 1.37%, and 1.69%, respectively. The degree of CO_2_ migration from the caprock to the overburden was considerably lower than that from the overburden of the goaf to the caprock. Most of the CO_2_ that diffused from the overburden of the goaf into the caprock is still effectively stored within the caprock.

**Table 4:** CO_2_ Migration in Different Strata at Different Pressures

	overburden of goaf to caprock migration			caprock to overburden migration			overburden to surface leakage		
time	10 MPa	15 MPa	20 MPa	10 MPa	15 MPa	20 MPa	10 MPa	15 MPa	20 MPa
1 year	1.88%	2.72%	3.29%	0.10%	0.15%	0.20%	0.00%	0.00%	0.01%
5 years	4.23%	6.03%	7.47%	0.47%	0.70%	0.93%	0.00%	0.01%	0.01%
10 years	5.63%	7.96%	9.88%	0.70%	1.03%	1.37%	0.01%	0.01%	0.02%
20 years	6.87%	9.61%	11.82%	0.87%	1.29%	1.69%	0.01%	0.01%	0.02%

The CO_2_ leaking from the overburden to the surface remained at an extremely low level. After 20 years under different pressures, the proportions of the total amount of CO_2_ diffused from the overburden to the surface were 0.01%, 0.01%, and 0.02%, respectively. Because the migration rate of CO_2_ toward the surface gradually approaches equilibrium over time, the 20 year leakage amount can be used to infer that the cumulative CO_2_ leakage from the goaf to the surface within 100 years would be less than 0.1%. According to the carbon capture and sequestration protocol under the California Low Carbon Fuel Standard and the IPCC scientific benchmark,^41,42^ a leakage amount of less than 1% within 100 years can be considered to indicate a low leakage risk and to meet the requirements for a high level of storage permanence. It can be considered that no significant leakage had occurred. At the same time, because the worst-case assumption was adopted in the initial condition setting, the numerical simulation conclusions were obtained under the field conditions with the highest leakage risk. Therefore, the leakage risk in actual engineering projects is expected to be lower than that predicted by numerical simulation. Therefore, the storage capacity of the target formation exceeds the highest-level requirements for CO_2_ geological storage under the relevant standards. On the basis of this, the relationship of difference in CO_2_ migration amount from the overburden of goaf to the caprock between different pressure intervals was calculated, as shown in Table 5. It was found that in the 10–15 MPa interval, the differences in the CO_2_ migration amounts at 1, 5, 10, and 20 years were 0.84%, 1.80%, 2.33%, and 2.74%, respectively. In the 15–20 MPa interval, the differences at the corresponding times were 0.57%, 1.44%, 1.92%, and 2.21%, respectively. Under the same pressure difference of 5 MPa, the migration amount difference was smaller in the higher-pressure interval (15–20 MPa). This indicated that for CO_2_ migration from the overburden of the goaf to the caprock, the growth rate of the CO_2_ migration velocity gradually decreased with increasing pressure.

**Table 5:** Difference in CO_2_ Migration from Overburden of Goaf to Caprock between Pressure Intervals

time	10–15 MPa	15–20 MPa
1 year	0.84%	0.57%
5 years	1.80%	1.44%
10 years	2.33%	1.92%
20 years	2.74%	2.21%

The curves of CO_2_ migration amount over time from the overburden of goaf to the caprock and from the caprock to the overburden under different pressures are plotted in Figure 7a. The figures showed that CO_2_ migration between different strata and under different pressures was most significant in the early stage of storage, especially the migration rate was fastest within the first five years. The migration amount within the first five years exceeded 50% of the total migration amount over 20 years. As time progressed, the CO_2_ migration rate gradually slowed down, and in the last five years, the migration amount no longer changed significantly. Under the three pressures, the migration amounts from the overburden of goaf to the caprock in the last five years were 7.1%, 6.5%, and 6.1% of the total 20-year migration amount, respectively. The migration amounts from the caprock to the overburden in the last five years were 7.4%, 7.2%, and 6.8% of the total 20 year migration amount, respectively, which were much lower than the migration amounts in the first five years. The CO_2_ migration in different strata all shows a trend of being faster at first and then slower. This pattern was consistent with the one obtained by of Wang and Peng^43^ using the phase-field method. This behavior was attributed to the interaction of multiple forces during geological storage. The force that promotes CO_2_ migration is the pressure difference, while the forces that resist included viscous force, capillary resistance, and gravity. At the interface between the overburden of goaf and the caprock, the capillary resistance was given by the following equation.

7 $$\Delta P_{c}=J\left(S_{w}\right)\times \sigma \times \cos\;\theta \times \left(\sqrt{\frac{\phi_{2}}{k_{2}}}-\sqrt{\frac{\phi_{1}}{k_{1}}}\right)$$

where *J*(*S*_*w*_) is the Leverett *J*-function; σ is the interfacial tension, N/m; θ is the contact angle; ϕ is porosity; *k* is permeability, md.

**Figure 7:**
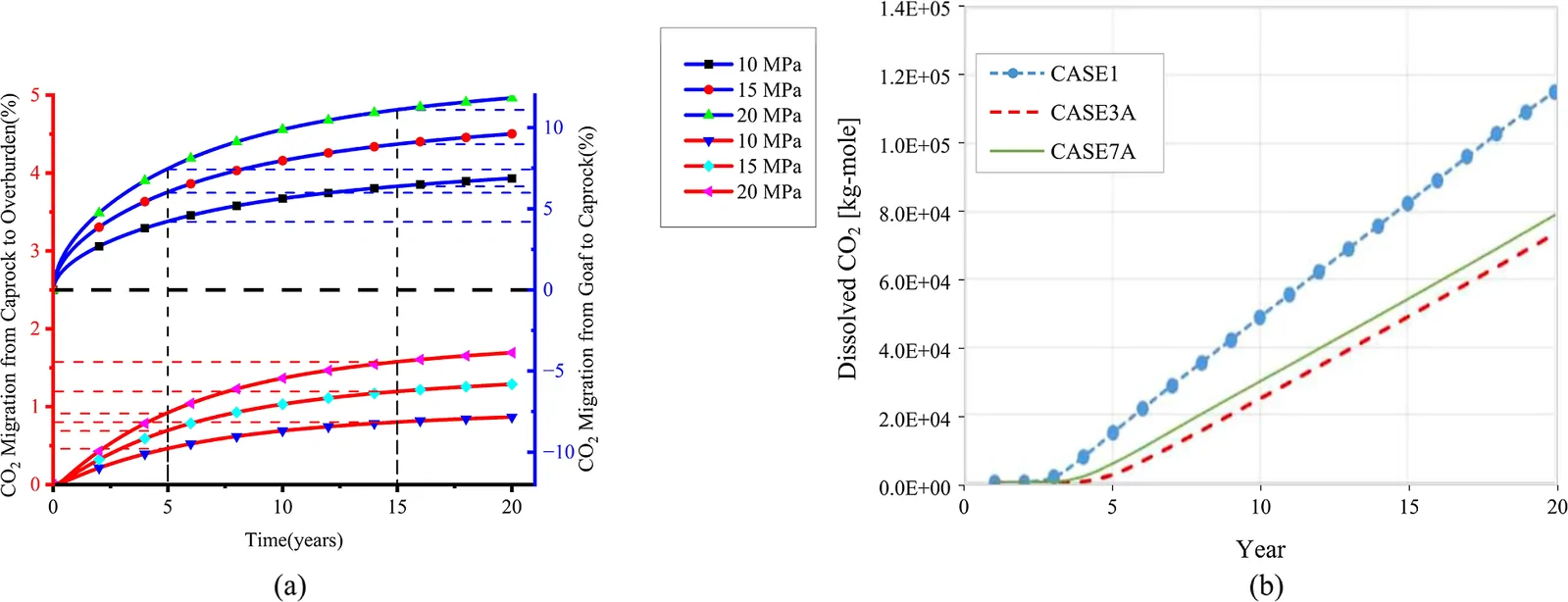
Temporal evolution of CO_2_ migration over time. (a) Curves of residual CO_2_ content in goaf-caprock system over time; (b) existing leakage curve of CO_2_ geological storage.

According to eq 7, in which the Leverett *J*-factor was taken as 0.4 for sandstone,^44^ the capillary resistance at the boundary between overburden of goaf-caprock and caprock-overburden was calculated to be 56.5 KPa and 0 KPa, respectively. The pressure differences and gravity forces in different regions at different times are shown in Table 6. The viscous force was calculated using the following formula

8 $$\tau =\mu \frac{du}{dy}A$$

where τ is the viscous force, N; d*u*/d*y* is the velocity gradient, s^–1^; *A* is the plate area, m^2^.

**Table 6:** Pressure Difference and Gravity in Different Regions at Different Times (KPa)

	overburden of goaf–caprock boundary			caprock–overburden boundary		
time	10 MPa	15 MPa	20 MPa	10 MPa	15 MPa	20 MPa
pressure difference at year 1	392.9	500.6	601.2	0	0	0
pressure difference at year 20	60.2	78.8	98.3	0	0	0
gravity	0.43	1.15	1.42	0.02	0.06	0.07

According to eq 8, based on the velocity gradient data extracted from the COMSOL software, the maximum viscous force under a storage pressure of 20 MPa was 2.3 × 10^–6^ KPa. This value was more than 3 orders of magnitude lower than pressure difference, capillary resistance, and gravity and therefore could be neglected.

Furthermore, the results of the present study were compared with those reported by Onoja and Shariatipour (Figure 7b).^45^ The conclusion based on the numerical simulation results is that CO_2_ migrates in the reservoir and the cap rock. The maximum migration of CO_2_ in the reservoir after 20 years is 6%, no leakage occurred, and the migration pattern of CO_2_ is generally consistent. This agreement supports the validity of the numerical simulation results.

At the overburden of the goaf–caprock boundary, the pressure difference in the first year was an order of magnitude higher than gravity and capillary resistance. At this stage, the pressure played a dominant role in CO_2_ migration. In the 20th year, the pressure difference decreased to the same order of magnitude as that of capillary resistance and gravity. These three forces competed with each other and jointly acted on CO_2_ migration, causing the CO_2_ migration rate to slow down. At the caprock-overburden boundary, both the pressure difference and the capillary resistance were 0 KPa. When the driving force for CO_2_ migration cannot effectively overcome the subsequent capillary resistance, migration nearly stops. This further confirmed the long-term stability of the CO_2_ geological storage. The simulation results clearly demonstrated that within the pressure range of up to 20 MPa and over a time scale of 20 years, this geological structure effectively prevented upward CO_2_ migration and no leakage to the surface had occurred. Therefore, they possess reliable geological conditions for implementing long-term CO_2_ geological storage.

### 3.2 The Temporal Evolution Characteristics of Surface Deformation

In order to investigate the surface deformation caused by the long-term storage of CO_2_ in the coal mine goaf after its injection. Based on the CO_2_ migration range in Section 3.1, this section takes into account the storage pressure and the influence of CO_2_ migration on surface deformation and studies the surface deformation situation over 20 years under three different storage pressures (10 MPa, 15 MPa, 20 MPa) after CO_2_ injection, as shown in Figures 8–10.

**Figure 8:**
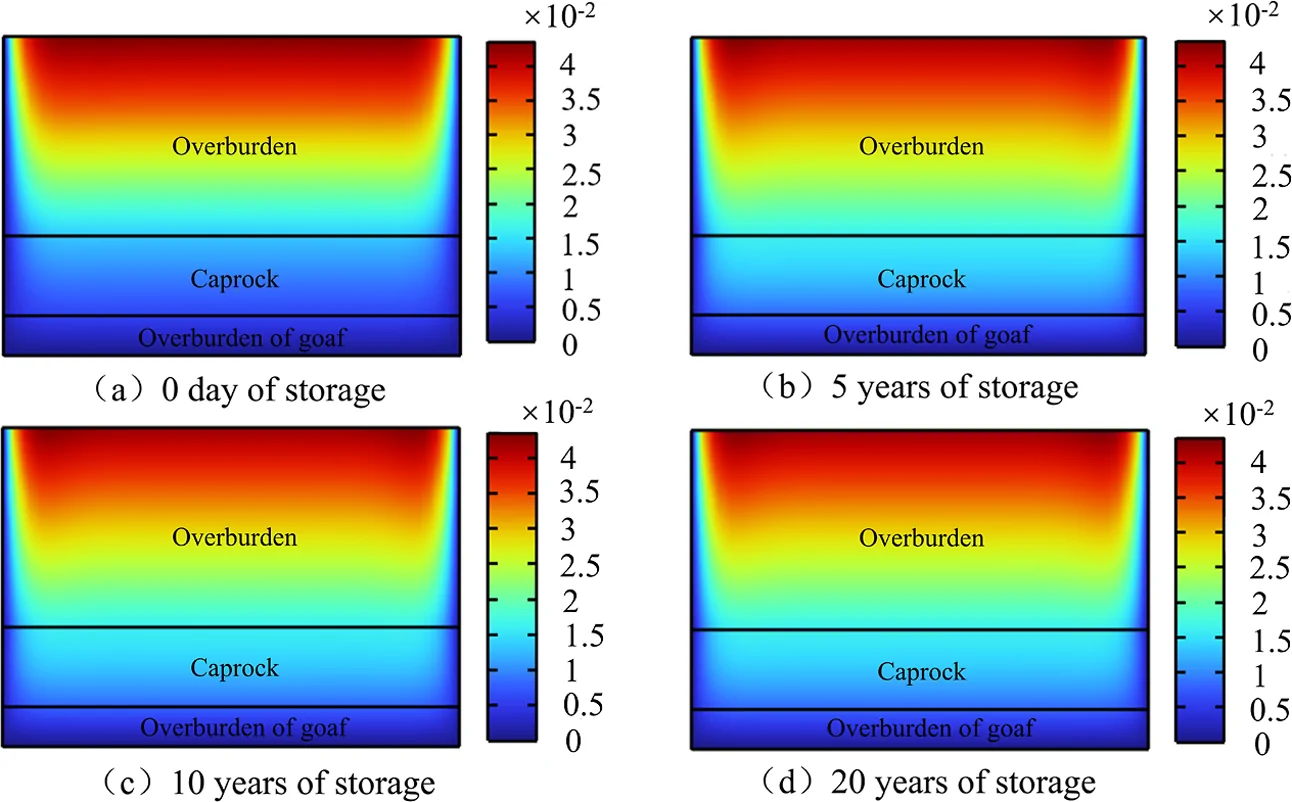
Surface deformation map of CO_2_ geological storage under a storage pressure of 10 MPa. (a) 0 day of storage; (b) 5 years of storage; (c) 10 years of storage; (d) 20 years of storage.

**9:**
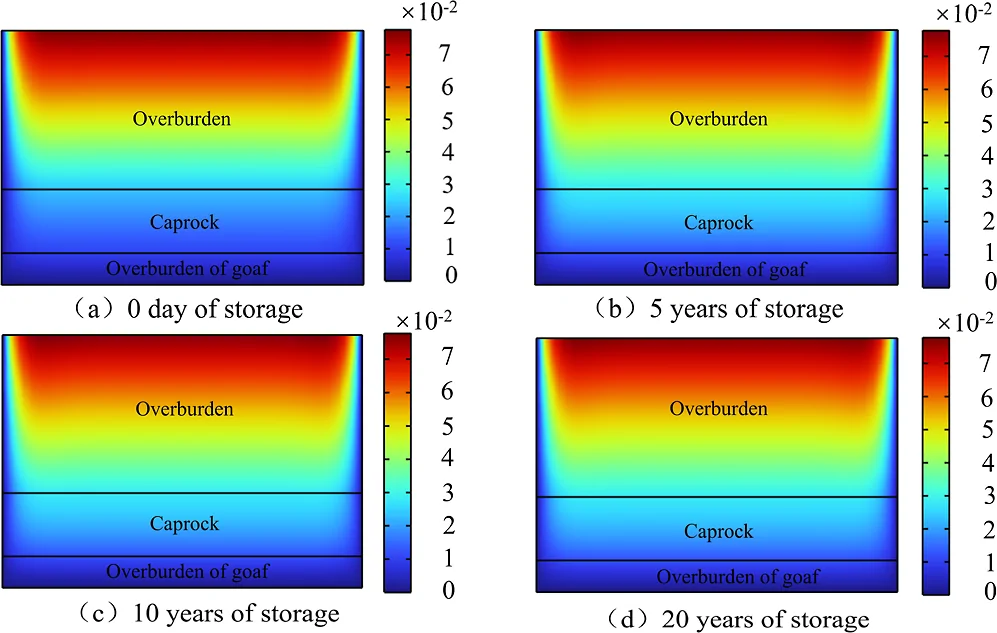
Surface deformation map of CO_2_ geological storage under a storage pressure of 15 MPa. (a) 0 day of storage; (b) 5 years of storage; (c) 10 years of storage; (d) 20 years of storage.

**Figure 10:**
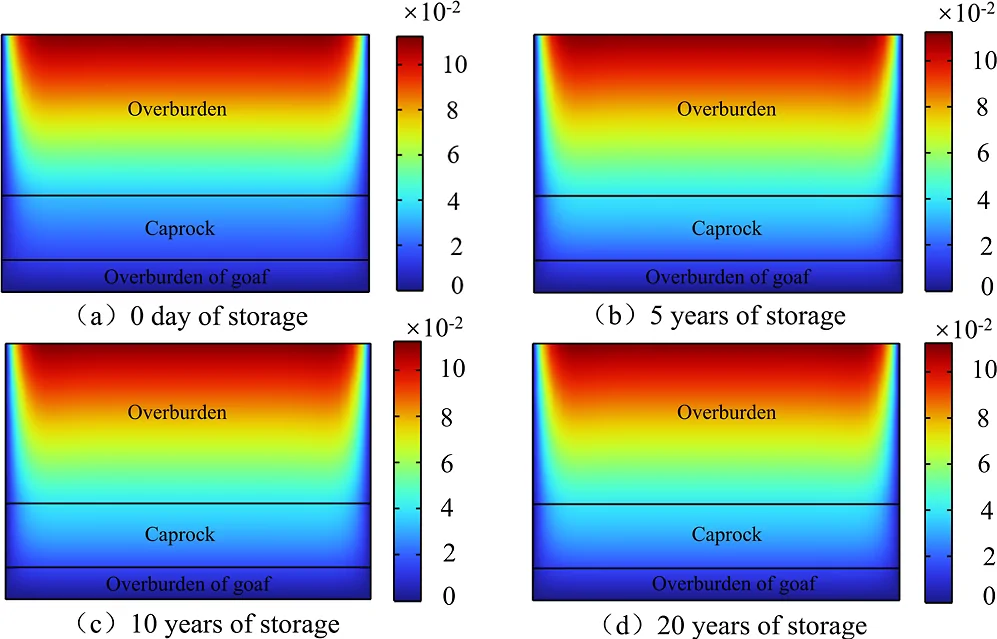
Surface deformation map of CO_2_ geological storage under a storage pressure of 20 MPa. (a) 0 day of storage; (b) 5 years of storage; (c) 10 years of storage; (d) 20 years of storage.

During the CO_2_ injection process, the amount of surface uplift continued to increase over time. When the CO_2_ injection is completed, the surface uplift reaches its peak.^46,47^ Figures 8–10a show the surface deformation at the initial storage state (0 days of storage) under storage pressures of 10, 15, and 20 MPa, respectively. At this point, it represents the initial storage state. As the storage time progresses, when it reaches 20 years, as shown in Figures 8–10b–d, the amount of surface uplift is gradually decreasing, presenting an overall pattern of initial uplift followed by a progressive decline from the peak-uplift position, while the surface remains above its preinjection level. Therefore, during the process of storage of CO_2_ in the coal mine goaf, the surface will undergo deformation. The surface uplift amount reaches its maximum value during the initial storage stage and then gradually decreases over time, eventually stabilizing.

According to the numerical simulation results, the surface uplift and the reduction from the peak uplift during the 20 year storage period under different sealing pressures are shown in Tables 7 and 8. Based on Table 7, the curves showing the changes of surface uplift over time under storage pressures of 10, 15, and 20 MPa are plotted, as shown in Figure 11. From the table and figure, it can be seen that the evolution process of surface deformation after storage of CO_2_ can be divided into three typical stages: (1) the first stage (rapid uplift-decline period, *t* = 0–1 year): during the initial stage, CO_2_ was fully injected. At this point, the amount of surface uplift reaches its maximum value. Under storage pressures of 10, 15, and 20 MPa, the surface uplift amounts were 0.060 m, 0.0917 m, and 0.123 m, respectively. After entering the storage stage, the surface uplift rapidly declined from the initial maximum value. After storage for 1 year, the surface uplift amounts under storage pressures of 10, 15, and 20 MPa were 0.0436 m, 0.078 m, and 0.1112 m, respectively, and the reductions from the peak uplift were 0.0164 m, 0.0137 m, and 0.011 m, respectively. The decline from the peak-uplift position was most pronounced during this stage. (2) The second stage (slow adjustment period, *t* = 1–2 years): the rate of decline in uplift decreases substantially. During the second year of storage, the incremental reductions in uplift during the second year are 8 × 10^–5^, 4 × 10^–5^, and 0 m under the pressure scenarios of 10, 15, and 20 MPa, respectively. Compared with the first stage, the incremental reductions in uplift have decreased substantially, and the system gradually transitions toward the long-term equilibrium state. (3) The third stage (stabilization period, *t* = 2–20 years): the displacement remains relatively stable, and the annual average change is no longer fluctuating, indicating that this stage has reached the final equilibrium state.

**Table 7:** Surface Uplift Amount after 20 years of Storage at Different Storage Pressures

initial storage pressure	storage surface uplift for 0 years (m)	storage surface uplift for 1 year (m)	storage surface uplift for 2 years (m)	storage surface uplift for 5 years (m)	storage surface uplift for 10 years (m)	storage surface uplift for 20 years (m)
10 MPa	6.00 × 10^–2^	4.36 × 10^–2^	4.352 × 10^–2^	4.351 × 10^–2^	4.351 × 10^–2^	4.351 × 10^–2^
15 MPa	9.17 × 10^–2^	7.8 × 10^–2^	7.796 × 10^–2^	7.795 × 10^–2^	7.795 × 10^–2^	7.795 × 10^–2^
20 MPa	1.23 × 10^–1^	1.112 × 10^–1^	1.112 × 10^–1^	1.11 × 10^–1^	1.11 × 10^–1^	1.11 × 10^–1^

**Table 8:** Incremental Reductions in Surface Uplift during Different Storage Intervals under Different Pressure Scenarios

initial storage pressure (MPa)	reduction in surface uplift from the peak value during the first year of storage (m)	reduction in surface uplift from the peak value during the second year of storage (m)	reduction in surface uplift from the peak value during the fifth year of storage (m)	reduction in surface uplift from the peak value during the 10th year of storage (m)	reduction in surface uplift from the peak value during the 20th year of storage (m)
10	1.64 × 10^–2^	8 × 10^–5^	0	0	0
15	1.37 × 10^–2^	4 × 10^–5^	0	0	0
20	1.1 × 10^–2^	0	0	0	0

**Figure 11:**
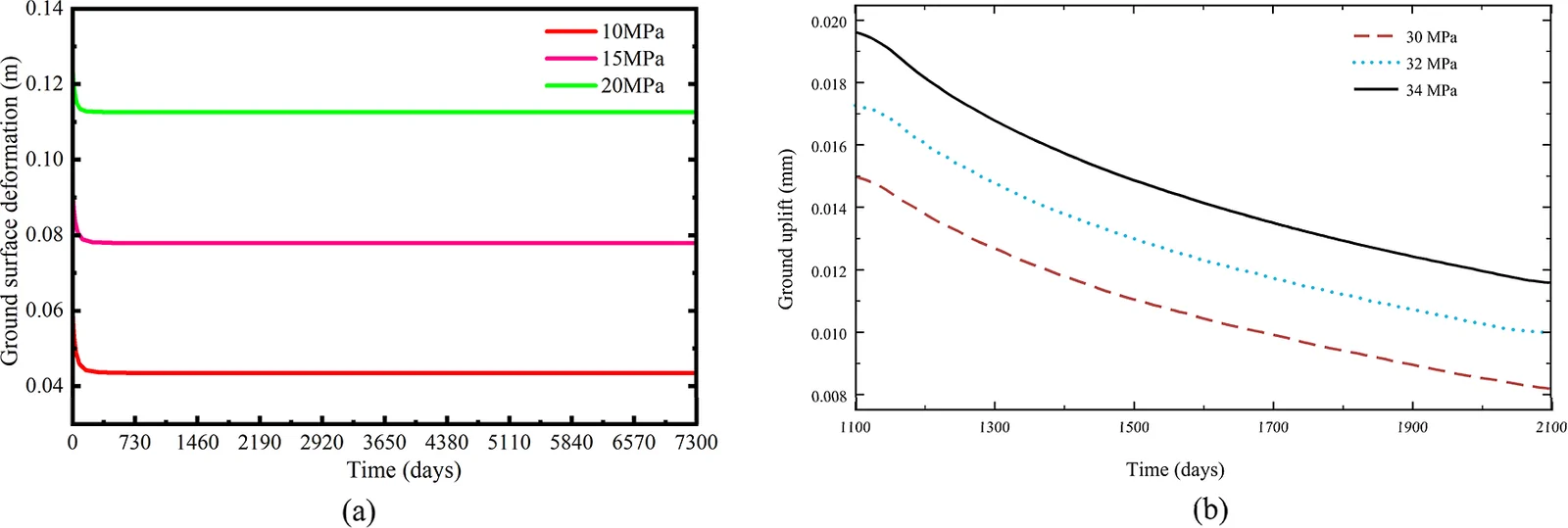
Surface deformation curves during CO_2_ storage: (a) surface deformation during the long-term postinjection storage period; (b) existing numerical simulation results of surface deformation.

Furthermore, the simulation results of this study (Figure 11a) were compared with those reported by Khan et al. (Figure 11b).^48^ The surface deformation patterns were found to be fully consistent, demonstrating the correctness of our numerical simulation results. They investigated CO_2_ storage in a depleted oil reservoir, whose geological structure, material properties, permeability characteristics, and boundary conditions differ from those of the coal mine goaf considered in this study. Consequently, the peak surface uplift and surface-uplift recovery ratios obtained in the two studies are different.

### 3.3 Evolution of the Pressure Field and Mechanism of Initial Uplift and Subsequent Decline

In order to investigate the mechanism governing the initial uplift and its subsequent decline from the peak-uplift position, the surface displacement and the evolution of pore pressure of the overburden strata were simultaneously monitored. The pore pressure of the overburden strata changed with time as shown in Table 9, and the time series diagram of deformation-pressure evolution was plotted as shown in Figure 12. The synchronous changes of these two factors clearly reveal the complete evolutionary process of the surface deformation from the initial nonequilibrium state to the final equilibrium state.

**Table 9:** Changes in Overburden Skeleton Pore Pressure with Time under Different Storage Pressures

initial storage pressure (MPa)	overburden pore pressure after being sealed for 0 years (MPa)	overburden pore pressure after being sealed for 1 year (MPa)	overburden pore pressure after being sealed for 2 years (MPa)	overburden pore pressure after being sealed for 5 years (MPa)	overburden pore pressure after being sealed for 10 years (MPa)	Overburden pore pressure after being sealed for 20 years (MPa)
10	4.27	0.59	0.572	0.57	0.57	0.57
15	4.34	0.678	0.659	0.656	0.656	0.656
20	4.41	0.752	0.745	0.744	0.744	0.744

**Figure 12:**
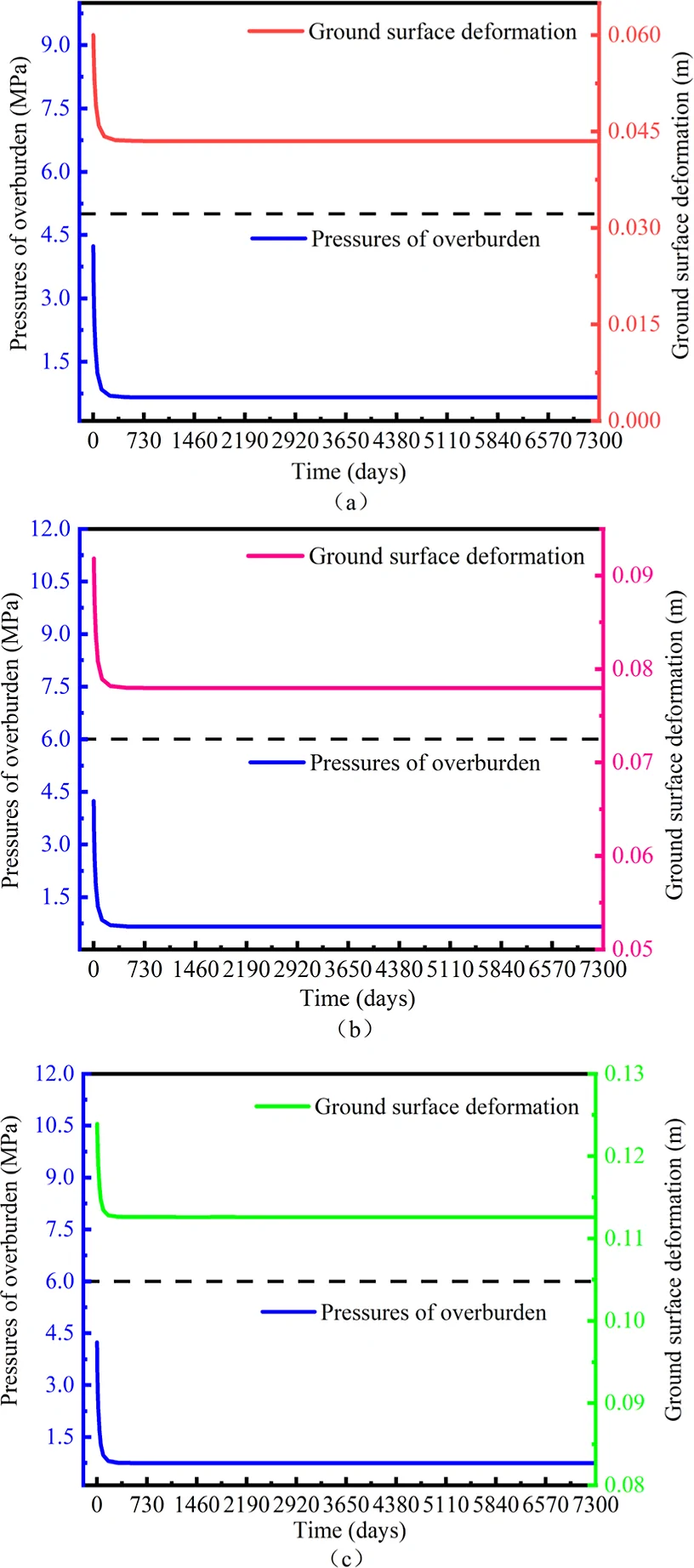
Surface deformation and strata pressure evolution under different storage pressures: (a) 10 MPa; (b) 15 MPa; (c) 20 MPa.

The image indicates that, although the storage pressures are different, the evolution of surface displacement shows a synchronous characteristic with the changes in the pore pressure.^49–52^ According to the effective stress principle (eq 4), it is found that the initial uplift and its subsequent decline from the peak-uplift position are governed by the continuous evolution of effective stress.

(1) Initial nonequilibrium state and peak (*t* = 0): In the initial stage, the pore pressure was the highest at 4.27, 4.34, and 4.41 MPa, respectively. At this time, the surface uplift reached its maximum at 0.060 m, 0.0917 m, and 0.123 m, respectively. According to the principle of effective stress, at this point, the effective stress borne by the rock skeleton of the overburden strata is the smallest. Therefore, the compressive deformation generated was the smallest. The surface was in the initial expansion stage, and the uplift of the surface reached a peak.
(2) Elastic rebound (from *t* = 0 to 2 years): after being in storage for 2 years, the pore pressures of the overburden strata are 0.572, 0.659, and 0.745 MPa, respectively. Compared with the initial nonequilibrium stage, the pore pressure is gradually decreasing, while the effective stress of the rock skeleton is gradually increasing. This causes the rock skeleton to undergo compressive deformation, and macroscopically, this is manifested as a decline in positive surface displacement from the peak-uplift position, although the surface remains above its preinjection level.
(3) Steady-state equilibrium (*t* = 2–20 years): After being stored for 20 years, the pore pressures of the overburden strata are 0.57, 0.656, and 0.744 MPa, respectively (Table 7). Compared with the elastic rebound stage, the pore pressure basically no longer changes and gradually stabilizes. The effective stress of the rock skeleton also gradually stabilizes, the surface hardly undergoes displacement anymore, and a final stable equilibrium state is reached.

### 3.4 Surface-Uplift Recovery Ratio under Different Storage Pressures

To further investigate the influence of storage pressure on the postpeak decline in surface uplift, the surface-uplift recovery ratios over the 20 year storage period were calculated for the different storage pressures. The surface-uplift recovery ratio is defined as the proportion of the peak surface uplift that has declined after n years of storage. This parameter represents the extent to which the surface has moved downward from its peak-uplift position toward the preinjection reference level. The Surface-uplift recovery ratio calculation is as shown in eq 9. The Surface-uplift recovery ratios are shown in Table 10.

9 $$R\left(n\right)=\frac{D_{\max}-D_{n}}{D_{\max}}\times 100\%$$

where *R*(*n*) represents the Surface-uplift recovery ratio, %; *D*_max_ denotes the maximum surface deformation, meters; *D*_*n*_ indicates the surface deformation in the *n*th year, meters.

**Table 10:** Surface-Uplift Recovery Ratio after 20 Year Storage under Different Storage Pressures

initial storage pressure (MPa)	surface-uplift recovery ratio after 1 year storage (%)	surface-uplift recovery ratio after 2 year storage (%)	surface-uplift recovery ratio after 5 year storage (%)	surface-uplift recovery ratio after 10 year storage (%)	surface recovery rate after 20 year storage (%)
10	27.33	27.47	27.48	27.48	27.48
15	14.94	14.98	14.99	14.99	14.99
20	9.59	9.59	9.76	9.76	9.76

As can be seen from Table 10, the surface-uplift recovery ratios after 1 year of storage at pressures of 10, 15, and 20 MPa are 27.33%, 14.94%, and 9.59%, respectively. The surface-uplift recovery ratio changes significantly in the first year of storage. Figure 13 shows that the surface uplift declines rapidly from its peak value during the first year. As the storage time progresses, starting from the second year, the surface-uplift recovery ratio subsequently increases slowly and eventually stabilizes. The surface uplift recovery ratios after storage for 20 years at pressures of 10, 15, and 20 MPa were 27.48%, 14.99%, and 9.76%, respectively. This indicates that the greater the storage pressure, the lower the surface-uplift recovery ratio. This is because the higher the initial storage pressure, the lower the effective stress borne by the rock skeleton, which subsequently triggers more surface deformation.^49^ However, the extremely high initial expansion state also led to a sharp increase in the effective stress during the recovery stage and an irreversible adjustment of the rock structure. Therefore, the surface-uplift recovery ratio decreased with the increase of the storage pressure, which is consistent with the mechanism governing the postpeak decline in uplift discussed in Section 3.3.

**Figure 13:**
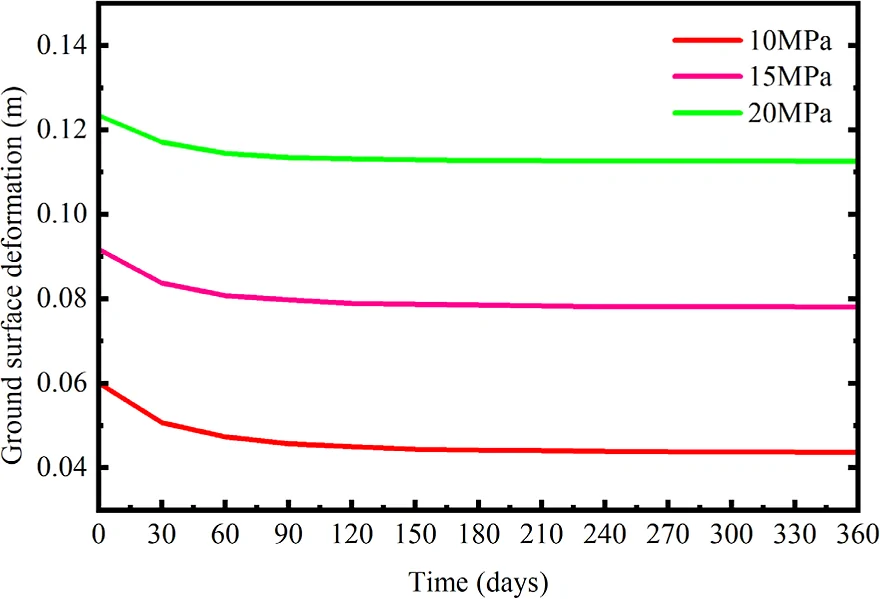
Surface deformation after one year of storage under different storage pressures.

In conclusion, in the practice of CO_2_ storage in coal mine goafs, it is essential to precisely control the storage pressure. This is not only related to the long-term stability of the storage but also a key measure for preventing ecological damage caused by excessive surface deformation.

### 3.5 Limitations and Future Work

The primary objective of this study is to investigate CO_2_ storage in coal mine goafs, with particular emphasis on potential CO_2_ leakage and surface deformation during the storage process. Accordingly, several simplifying assumptions were adopted in the model: CO_2_–water–rock geochemical reactions were not considered, isothermal conditions were assumed, and the porosity and permeability of each geological unit were kept constant. These assumptions help clarify the relationships among multiphase flow, pore-pressure dissipation, effective stress, and surface deformation. Specifically, the following physical and chemical processes were simplified or excluded from the present model:

1. The rock pore structure may be modified, and its mechanical properties may be degraded by mineral dissolution induced by CO_2_–water–rock geochemical interactions. Consequently, the stability of CO_2_ storage in the goaf may be affected.
2. The permeability of each geological unit was assumed to be constant throughout the simulation. In an actual goaf, rock compaction, fracture opening or closure, stress redistribution, and chemical alteration may all cause permeability to evolve over time and therefore affect the predicted extent and rate of CO_2_ migration.
3. The simulations were conducted under isothermal conditions at a formation temperature. In practical applications, different geological formations have different temperatures. Heat transfer and pressure variations can affect the density, viscosity, and phase state of CO_2_ and may also induce thermal expansion or contraction of the surrounding rock, thereby altering CO_2_ migration and surface deformation.

Therefore, future studies should investigate the effects of CO_2_–water–rock geochemical reactions, stress- and damage-dependent permeability evolution, and nonisothermal processes on CO_2_ migration and surface deformation.

## 4 Conclusions

(1) CO_2_ migration is pressure-dependent and exhibits a distinct temporal pattern. Higher storage pressure leads to a larger migration range and faster initial migration rate. The migration process is most active in the first five years, accounting for over 50% of the total 20 year migration, after which it nearly ceases due to the equilibration of driving forces (pressure difference) and resisting forces (capillary resistance and gravity). Notably, even under a high storage pressure of 20 MPa, the cumulative CO_2_ leakage to the surface after 20 years remains negligible (0.02%), confirming the excellent sealing capacity of the caprock.
(2) Surface deformation is characterized by an initial uplift followed by a progressive decline from the peak-uplift position, while the surface remains above its preinjection level. The process can be divided into three stages: a rapid uplift-decline period (0–1 year), a slow adjustment period (1–2 years), and a stabilization period (2–20 years). The peak surface uplift occurs at the initial stage of storage, directly correlated to the storage pressure.
(3) The deformation mechanism is governed by effective stress changes. At the initial storage stage, a high pore pressure minimizes effective stress, leading to maximum surface uplift. As storage time increases, pore pressure dissipates and effective stress rises, causing compression of the rock skeleton and a subsequent decline in positive surface displacement from the peak-uplift position, without producing net subsidence relative to the preinjection surface. The synchronous evolution of the pore pressure and surface displacement validates this mechanism.
(4) The storage pressure inversely affects surface recovery rates. After 20 years, the surface recovery rates under storage pressures of 10, 15, and 20 MPa were 27.48%, 14.99%, and 9.76%, respectively. Higher storage pressures lead to a more intense initial expansion and a lower final recovery rate, indicating that precise pressure control is crucial for maintaining the long-term surface stability and storage safety.
(5) The target geological structure demonstrates a long-term storage suitability. The numerical simulation results confirm that under the investigated conditions (pressures up to 20 MPa over 20 years) the caprock effectively prevents upward CO_2_ migration, with no significant leakage to the surface. These findings provide a theoretical basis and scientific evidence for the safe and stable implementation of long-term CO_2_ geological storage in goaf environments.

## Data Availability

The data sets supporting the findings of this study are available within the article. Further reasonable requests regarding data can be made to the corresponding authors.
